# Demographic and Geographic Characteristics Associated with the Type of Prescription and Drug Expenditure: Real World Evidence for Greece During 2015–2021

**DOI:** 10.3390/healthcare12222312

**Published:** 2024-11-19

**Authors:** Georgios Mavridoglou, Nikolaos Polyzos

**Affiliations:** 1 Department of Accounting and Finance, School of Management, University of the Peloponnese, 24100 Kalamata, Greece; 2 Medical School, Democritus University of Thrace, 68100 Alexandroupoli, Greece; npolyzos@med.duth.gr

**Keywords:** pharmaceutical expenditure, drug consumption, cluster analysis, geographical prescription pattern, demographic differences and no demographic differences

## Abstract

Aim: Electronic prescribing has allowed for the collection of prescription data in real time in Greece for the first time. Hence, the aim of the current study was to present the characteristics of prescriptions for the Greek population during the period from 2015 to 2021. Methods: This retrospective study was based on data extracted from the nationwide Greek electronic prescription database between January 2015 and December 2021. Descriptive statistics methods were used for the needs of the study. As the basic figures examined depend on the size of the population, in order for the results to be comparable, we estimated the corresponding measures per inhabitant, using population data from the Greek Statistical Authority. Appropriate indicators for the comparison of consumption and expenditure over time were estimated. A study of the trend was also carried out using time series and linear regression models. In order to facilitate the design and implementation of specialized policies, it is useful to identify the drug categories with the highest consumption and expenditure, as well as the geographical areas that present similar characteristics. For the first, ABC analysis was used, which helps to identify the most popular categories of drugs, while for the second, cluster analysis was carried out. Agglomerative clustering was used to divide the regions into similar groups. This hierarchical clustering algorithm classifies the population into several clusters, with areas in the same cluster being more similar, and areas in different clusters being dissimilar. The Ward linkage method with Euclidean distance was used. Results: The analysis of prescription drug consumption and expenditure from 2015 to 2021 revealed significant fluctuations and trends across various drug categories, age groups, and geographical areas. Notably, the quantity of prescriptions increased by 20% since 2015, while expenditure surged by over 30%, with significant spikes following the end of the MoU in 2019 and the onset of the pandemic in 2020. In terms of expenditure, antineoplastic and immunomodulation agents (category L) held the largest share, driven by the introduction of new, costly drugs. The expenditure per inhabitant revealed gender and age disparities, with older populations, particularly women, incurring higher costs. Geographically, drug expenditure, and consumption varied significantly, with distinct regional clusters identified. These clusters, while showing some overlap in consumption and expenditure patterns, also highlighted unique regional characteristics. Conclusions: The insights into prescription drug consumption and expenditure trends offer a valuable basis for developing targeted interventions aimed at optimizing healthcare resource allocation. Moreover, the findings underscore the importance of addressing regional and demographic disparities in pharmaceutical use, thereby contributing to more equitable and cost-effective healthcare strategies. More specifically, the age distribution of prescriptions shows the increase in younger ages, which, as a result, anticipates the overall increase in prescriptions. The knowledge of the most convex categories of medicine, as well as the percentages of the use of generic drugs, shows where interventions should be made, with financial incentives and information through new information channels. The geographic disparities recorded should lead to policies that help the residents of hard-to-reach areas to access prescriptions. In addition, the present study provides a strategic framework for policymakers and healthcare managers to guide future studies and inform decision-making processes.

## 1. Introduction

Drugs are an essential and indispensable tool for patients’ prevention, diagnosis, treatment, and rehabilitation. For a rational use of drugs, patients must receive medications appropriate to their clinical needs, in doses that meet their requirements, for an adequate period, and at the lowest cost to them and the community, either through states or social insurance sickness funds [1,2]. Prescribing medications is one of general practitioners’ most critical therapeutic activities, and the quality of such practices is a relevant issue [3]. Therefore, the ultimate goal of a rational medical prescription is to optimize the therapeutic results, while also keeping in mind the individual characteristics of the patients, such as sex and age differences [4,5].

Demographic and geographic characteristics significantly influence the type of prescriptions and drug expenditure [1,3,6,7,8]. Studies have shown that factors such as age, gender, income, and education level play crucial roles in determining the types of medications prescribed and the overall spending on drugs [3]. Geographically, there are notable variations in prescription patterns and drug costs [9]. Urban areas often have higher prescription rates for certain medications compared to rural areas, possibly due to better access to healthcare facilities and specialists. Additionally, socioeconomic status and regional healthcare policies can lead to disparities in drug utilization and spending [1,3,6,8].

During Greece’s economic crisis (2010–2019), the primary goal of drug policy was to limit pharmaceutical expenditure to 1% (from 2%) of GDP (N 4336/2015—official government gazette 94/A/14.8.2015) within a total public expenditure cap of 6% [10]. Key measures included drug price reductions, the reintroduction of a positive list based initially on cash flow, mandatory discounts, and forced returns (claw backs) [11,12]. The pricing process has changed several times between 2010 and 2021 [12]. According to the latest legislative framework (N 4638/2019), the process is carried out once a year, and a revised price bulletin is issued every December with drug prices based on the average of the two lowest Eurozone prices, while the product should be priced in at least three Eurozone countries [10,13].

During the first memorandum of understanding (MoU) in 2010 to 2011 [14], Greece implemented significant structural changes in its healthcare system, including the consolidation of social health insurance funds into the EOPYY, the introduction of electronic prescription systems, and the integration of therapeutic protocols [15]. These reforms enabled the collection of prescription data and allowed the insured to choose pharmacies and opt for original drugs, instead of a generic one, by covering the cost difference. An impulse to expand the use of generic drugs aimed to reduce healthcare expenditure faced strong opposition from medical associations. Despite initial setbacks, the Ministry of Health announced that the prescriptions of active substance would be mandatory and that pharmacists must dispense the cheapest generic version. Again, this was amended due to the opposition of the doctors. Under the amendment, doctors are allowed to prescribe a specific “brand”, in which case patients will have to pay the price difference (if any) between the prescribed “brand” and the price of the generic alternative. This simply caused costs to shift from the public system to patients.

The end of the economic crisis and the period of the MoU coincided with the start of the pandemic, which put even more pressure on pharmaceutical spending but also led to two prolonged periods of quarantine: the first rigorous one in May 2020 and the second at the end of 2020 and the beginning of 2021 [14,16,17]. As a consequence of this period, the average copayments increased by 10% for pharmaceutical expenditure.

It should be noted that several studies extensively investigate the health policies implemented during the economic crisis and their economic outcomes in Greece [18,19,20,21,22]. Although, as mentioned above, the study of prescription characteristics is an important factor for policy making. In Greece, very few studies have been published on this specific topic; there have been some on out-of-hospital prescribing [23,24] and some on in-hospital [15,25] prescribing. The reason for this is mainly due to difficulty in accessing the data.

This research focuses on the characteristics of out-of-hospital prescribing. Analytically, the prescription is analyzed by ATC-1 and -2 categories and examined by gender and age group. The main ATC-1 categories are identified in terms of quantity and cost, and the use of generic drugs is examined. Trends are analyzed according to historical data.

## 2. Materials and Methods

### 2.1. Data

In this postdoc study, we retrospectively analyzed the existing electronic health record database of the e-Government Center for Social Security Services prescription database (IDIKA), which covers almost 100% of the country’s population. The e-prescription network has been available since 2011 and is the only pathway enabling the prescription of medications that can be reimbursed by the National Organization for the Provision of Health Services (EOPYY). The Minister of Digital Governance exercises the supervision and control of the IDIKA. The e-prescription includes data for the period 2015–2021, with the following distinguishing variables: therapeutic category at ATC level (level 1 to 4), year of prescription, gender and age of the patient, type of drug, geographic region, physician speciality, and the quantity variables, value, payment by the insurance agency, number of prescriptions.

The present study of the characteristics of prescription data from 2015 to 2021 is an update of a relevant Democritus University of Thrace (DUTh) research [23].

The necessary demographic data for Greece were obtained from the Hellenic Statistical Authority.

### 2.2. Statistical Analysis

The analysis was divided into three parts. The first one included a descriptive overview of the per capita gross pharmaceutical expenditure (expressed in EUR) and drug consumption per capita, using the Greek Statistical Authority’s estimations for the population by age and year. The gross estimations were calculated as:

$$E_{pc,\;t}=\left[E_{t}/(P_{t})\right]$$

$$V_{pc,\;t}=\left[V_{t}/(P_{t})\right]$$

where $E_{pc,\;t}$ is the expenditure per capita at year *t*, $V_{pc,\;t}$ is the volume of drug consumption per capita, $E_{t}$ is the expenditure at year *t*, $V_{t}$ is the volume of drug consumption at year *t*, and $P_{t}$ is the estimation of the population at year *t*.

The annual change in expenditure (${\Delta E}_{t}$) and volume (${\Delta V}_{t}$) and per capita expenditure (${\Delta E}_{pc,\;t})$ and volume ${(\Delta V}_{pc,t})$ is given by the formula:

ΔQt=(Qt+1−Qt)Qt

where: $Q_{t}\;can\;be\;E_{t},\;V_{t},\;and\;then$ called annual rates, or ${{\;V}_{pc}}_{t}{,\;E_{pc}}_{t}\;and$ called change rates.

ABC analysis was used to determine the importance of each ATC-1 group to prescription. ABC analysis is a managerial tool that determines the value of the items based on their importance to the business. ABC ranks items on demand and cost, and groups items into classes based on those criteria. This helps leaders understand which products are most critical to the financial success of their organization. For the implementation of the ABC analysis, the total expenditure and consumption of each ATC-1 category was calculated for the period 2015–2021, and the relevant percentages were calculated.

As the volume and value of prescription drugs depend on the population of each period, we estimated an index, which compares the prescription of period t with a reference year (set in 2015). The index was calculated as a price index, as follows [26,27]:

$$I_{t}=\left[\left(\frac{E_{t}}{E_{0}}\right)/(\frac{V_{t}}{V_{0}})\right]\cdot 100$$

To study the impact of age and gender on pharmaceutical expenditure, the deviation of the average expenditure and consumption of each subgroup from the average expenditure of the population was estimated using the following formula:

$$\frac{{Mean\;value}_{x}^{G}\;}{Mean\;value}\cdot 100$$

where *x* is the age group (0–14, 15–29, 20, 44, 45–59, 60+), and *G* is the gender (male, female).

The second part includes trend analysis using linear regression models to answer the question: “What is likely to happen?”.

The third part includes cluster analysis to examine similarities between regions in pharmaceutical consumption and expenditure. Cluster analysis is a method frequently used in the literature on healthcare data [28,29]. Theodorides et al. [30] present a detailed description of cluster analysis. The technique is used in case disease classification [31,32] and healthcare system classifications [33,34]. For our research purposes, an agglomerative (bottom-up) hierarchical clustering algorithm is used to divide the regions into similar groups. This is a hierarchical clustering algorithm that classifies the population into several clusters, with areas in the same cluster being more similar and areas in different clusters being dissimilar. According to the 2021 data, two data vectors for each region were created. The first includes the mean quantity per capita by anatomical therapeutic chemical (ATC)-1 group and gender, and the second is the mean value per capita by ATC-1 group and gender. The Ward linkage method with Euclidean distance was used. Cophenetic correlation coefficient (CCC) has been used to verify the cluster quality [35,36]. The analysis of the results shows the different patterns of pharmaceutical consumption and expenditure in the country, while comparing the results of the two-cluster analysis can identify possible differences in prescription policies between the regions.

IBM Statistics V.29.0.0 was used for statistical analysis and clustering analysis, and CCC calculations were performed using statistical program R v4.4.2.

## 3. Results

### 3.1. Total Consumption and Expenditure

Table 1 shows the total prescription consumption fluctuation (in quantity and expenditure). The quantity increases every year and in 2021 was 20% more than in 2015. The annual change in consumption per capita also shows a significant yearly increase, between 2.4% to 4.9%. On the expenditure side, only 2016 showed a decrease, with the following years presenting a significant increase. Compared to 2015, the 2021 expenditure showed an increase of over 30%, and there was also a significant yearly increase in expenditure per capita, especially after the end of the MoU (2019) and the pandemic (2020).

**Figure 1:**
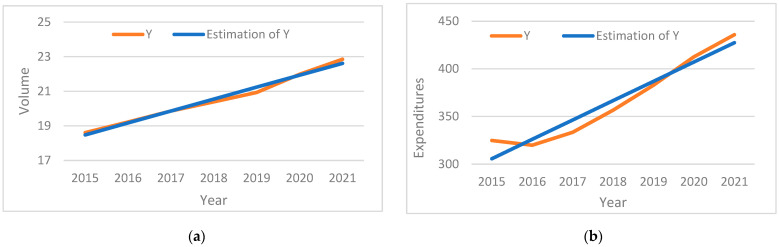
Linear trend analysis, (**a**) volume; (**b**) expenditure.

Table 2 and Figure 1 present the trend analysis of the volume and the expenditure per capita. The coefficient for the volume per capita is statistically significant (*p*-value < 0.001) and positive, with a value of 0.69 (95%CI: 0.60–0.79), which leads to the conclusion that the mean drug consumption will increase every year by 0.69. The coefficient for the expenditure per capita is statistically significant (*p*-value < 0.001) and positive, with a value of 20.29 (95%CI: 14.21–26.38), which leads to the conclusion that yearly expenditure per capita will increase by 4.5% from 2021’s mean yearly expenditure per capita (EUR 20.29).

### 3.2. Pharmaceutical Characteristics

**Figure 2:**
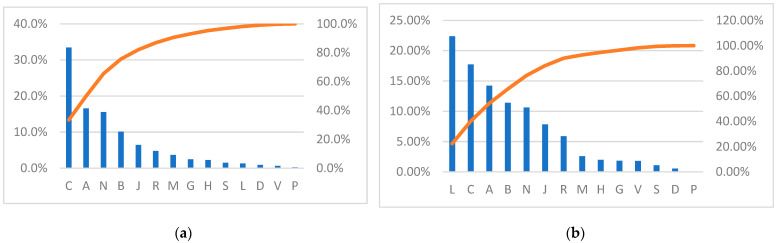
ABC analysis; (**a**) for quantity and (**b**) expenditure by ATC-1 categories, 2015–2021. (A: alimentary tract and metabolism; B: blood and blood-forming organs; C: cardiovascular system; D: dermatology; G: genito-urinary system and sexual hormones; H: systemic hormonal preparations; J: anti-infectives for systemic use; L: antineoplastic and immunomodulating; M: musculo-skeletal system; N: nervous system; P: antiparasitic products; R: respiratory system; S: sensory organs; V: various).

Figure 2 presents an ABC analysis of quantity and expenditure. As for the quantity, the drugs in category C (cardiovascular system) were double the consumption of the following categories: A—alimentary tract and metabolism, N—nervous system, B—blood and blood-forming organs. These four drug groups covered 80% of the total volume. In the annual distribution of the drug volume, group C occupied more than 30%; although, this percentage decreased from 36% in 2015 to 31.3% in 2021.

Taking into account total expenditure, category L (antineoplastic and immunomodulating agents) had the largest share of the expenditure (22.4%), followed by category C (cardiovascular system), with a percentage of 17.7%, and category A (alimentary tract and metabolism), with a percentage of 14.2%. The expenditure for these three categories exceeded 50% of the total spending (53.3%). They were followed by two other categories with a percentage of over 10%: B (blood and hematopoietic organs) with 11.4% and N (nervous system) with 10.6%.

**Figure 3:**
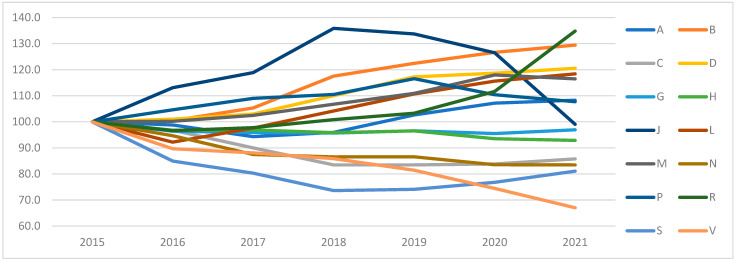
Ratio (expenditure/quantity) per year and ATC-1 category (A: alimentary tract and metabolism; B: blood and blood-forming organs; C: cardiovascular system; D: dermatology; G: genito-urinary system and sexual hormones; H: systemic hormonal preparations; J: anti-infectives for systemic use; L: antineoplastic and immunomodulating; M: musculo-skeletal system; N: nervous system; P: antiparasitic products; R: respiratory system; S: sensory organs; V: various).

The fluctuation of quantity and expenditure over time is significant. The expenditure/quantity ratio was calculated to compare the rate of change in quantity and expenditure of each category. Figure 3 presents, in a timeline chart, the index $I_{t}^{K}=\left[\left(\frac{E_{t}}{E_{2015}}\right)/(\frac{V_{t}}{2015})\right]\cdot 100$, where *t* is the year, and *K* is the ATC group, with 2015 as the base where all ATC groups have index value = 100.

The following ATC-1 categories show a ratio of less than 100: G (genito-urinary system and sex hormones), H (systemic hormonal preparations, excluding sex hormones and insulins), C (cardiovascular system), N (nervous system), and S (sensory organs). Although the quantities increased, expenditure increased at a lower rate, so costs were decreasing. This may be due to the increase in generic drug consumption, the end of the protection period of original drugs, or the appearance of lower priced drugs. In contrast, for category L (antineoplastic and immunomodulating agents), the rate of change in expenditure exceeded the rate of change in quantity, possibly due to the appearance of new, more expensive drugs.

For the seven years 2015–21, the percentage of the contribution of generic drugs compared to the total expenditure on prescription drugs (Table 3) reached 16%. The five anatomical groups with the highest contribution of generic drugs are category D (dermatology), followed by the anatomical category C (cardiovascular system), showing an increase of 9%, N (nervous system) with a rise of 9%, M (musculoskeletal system) with a decrease in generic drugs of 5%. For ATC-1 category C (cardiovascular system), the generic use increased continuously, from 24.1% of total spending in this category to 33.4%, which explains why expenditure was reduced, despite the increase in prescription volume. On the contrary, in category L, the percentage of expenditure from the prescription of generic drugs remained close to 4.5% to 5%, from the lowest percentages of generic drugs. A concluding result is a possible failure of the generic marketing to these patients.

### 3.3. Demographic Characteristics

**Figure 4:**
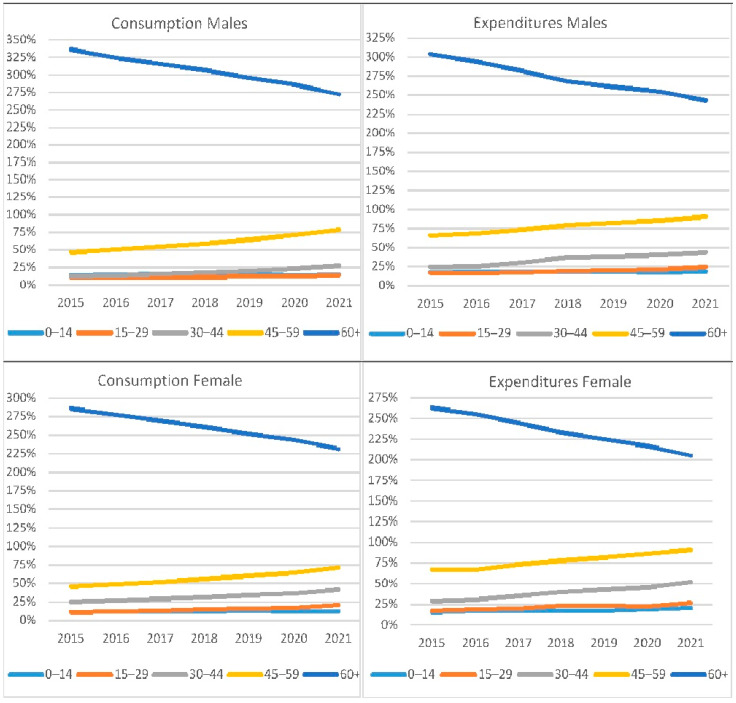
Consumption and expenditure per capita by sex and age group, as a per cent of mean, 2015–2021.

Considering gender and age group, Figure 4 shows the deviation of the pharmaceutical consumption and expenditure per capita by year, age group, and gender, as a per cent of the mean consumption and spending. For men, the consumption per capita varied from 16.7 to 20.6 with an annual increase in rates from 3.6% (2016) to 3.4% (2021). The expenditure per capita varied from EUR 307 (2015) to EUR 426 (2021), with rates increasing from 0.3% to 5.4%. For women, the consumption per capita varied from 20.4 (2015) to 25.6 (2021), with an annual increase in rates from 3.0% (2016) to 4.4% (2021). The expenditure per capita varied from EUR 342 (2015) to EUR 446 (2021), with rates increasing from −3.1% (2016) to 8.0% (2020) and 5.9% (2021). Drug consumption and expenditure for all age groups, except 0–14, were greater for women than men, but as age increased, the difference between genders reduced. For the older men (age group 60+), the deviation from the mean value decreased from 336% (2015) to 272.2% (2021) for consumption and from 304% to 243% for expenditure. Corresponding to women, the average price of the quantity changed from 236% in 2015 to 231% in 2021. The spending percentages were 263% in 2015 and 205% in 2021. The annual change rate increase in both measures was much lower than the other groups; although the absolute numbers increased, the percentages decreased.

As mentioned above, participation in the expenditure and consumption of medicines for the 60+ age group was much higher than the other age groups. This is why the participation rates of this age group were high in the distribution of consumption (78.1%) (Table 4a) and expenditure (75.9%) of generic drugs by age (Table 4b), even though it has been slightly reduced in the last seven years. Of course, this particular group was not the highest in the use of generic drugs, as 27.6% of the consumption and only 17.4% of the total expenditure concerned generic drugs. Of course, this percentage was the highest spending on generic drugs among the age groups.

Analyzing the prescription for the age group 0–14 at the ATC-2 level, three categories (J01 (antibiotics), J07 (vaccines), and R03 (medicines for obstructive airways disease)) had the most significant volumes. On the expenditure side, therapeutic group J07 (vaccines) accounted for the most critical proportion, approaching 70% of total prescription expenditure for ages 0–14, also showing an increase in per capita expenditure of around 40%.

For the 15–29 age group, most of the volume of drugs belonged to the categories: J01 (antibiotics), B03 (drugs against anemia), J07 (vaccines), R03 (drugs for obstructive diseases of the airways), and N03 (anticonvulsants). On the expenditure side, therapeutic groups L04 (immunosuppressive agents) and J07 (vaccines) accounted for this age group’s highest per capita amount. It is worth noticing that the significant expenditure of the J07 category was also due to the large volume of vaccines; on the contrary, the therapeutic category L04 did not have a large volume but a considerable treatment cost. The therapeutic group L04 also showed a significant increase from 2015 to 2021.

According to these results, for the 30–44 age group, most of the drug volume belonged to the categories: B03 (antianemia drugs), J01 (antibiotics), N05 (psychotropic drugs), A12 (mineral supplements), B01 (antithrombotics), and N06 (psychoanalgesics). The above categories covered 50% of the drug volume. On the expenditure side, the three therapeutic groups from group L (antitumor and immunomodulatory agents) were in the top six, according to per capita expenditure. Therapeutic groups L04 (immunosuppressants), L01 (antineoplastic drugs), L03 (immunostimulatory agents), B03 (antianemia drugs), J05 (antivirals), N05 (psychotropics), had the highest per capita amount for this age group. The above therapeutic groups, except L03, significantly increased from 2015 to 2021. The consumption of N05 (psychotropics) could be due to economic and pandemic crises. It should be noticed that unemployment rate increased over 20% during the previous decade and even more in these ages.

For the age group 45–59 years old, the ATC-2 categories with a higher drug consumption were C10 (hypolipidemic agents), N05 (psychotropic agents), C09 (antihypertensive agents), A10 (drugs for diabetes mellitus), N06 (psychoanalgesics), J01 (antibiotics), A02 (antacids), and B01 (antithrombotics). The above categories covered more than 50% of the total drug consumption. On the expenditure side, the therapeutic groups L04 (immunosuppressive agents) and L01 (antineoplastic drugs) were the ATC-2 categories with the highest average expenditure per capita, followed by A10 (drugs for diabetes mellitus), C10 (hypolipidemic agents), and N05 (psychotropic drugs). All the above therapeutic groups showed a significant increase from 2015 to 2021, and the average per capita expenditure difference for the same therapeutic groups between age groups is noticeable. A typical example is group L04, with an average per capita expenditure of EUR 29.1 for the 30–44 age group, compared to EUR 52.6 for the 45–59 age group.

Finally, analyzing the prescription data for the age group 60+, most of the drug volumes belonged to the following ATC-2 categories: C09 (antihypertensive agents), A10 (drugs for diabetes mellitus), B01 (antithrombotics), A02 (antacids), C07 (B-receptor blockers), N05 (psychotropics), and N06 (psychoanalgesics). The above categories covered 60% of the drug volume. These categories were similar to the categories of the 45–59 age group. On the expenditure side, therapeutic group L04 (immunosuppressants) fell to sixth place, with a higher average per capita expenditure for A10 (drugs for diabetes mellitus), L01 (antineoplastic drugs), C10 (hypolipidemic agents), C09 (antihypertensive agents), and B01 (antithrombotics).

### 3.4. Geographic Characteristics

Table 5a,b presents pharmaceutical consumption (Table 5a) and expenditure per capita (Table 5b) by ATC and geographical area. According to the results, the groups with the highest expenditures were L—antineoplastic and immunomodulating (22.5% of the total), C—cardiovascular system (17.5% of the total), and A—alimentary tract and metabolism (12.2% of the total). Geographically, the coefficient of variation ranged between 6% and 40%; for only three (A, V—various, and J—anti-infective for systemic use), the value was less than 10%. The regional consumption was EUR 327 per capita, ranging from EUR 278 in the South Aegean region to EUR 345 in Western Greece.

The same findings were also found for consumption: C—cardiovascular system, A—alimentary tract and metabolism, N—nervous system, and B—blood and blood-forming organs had the highest values. The accumulated consumption of these four groups exceeded 80% of the total consumption. Geographically, there was quite a high variability, with a coefficient of variation of about 20%.

### 3.5. Similarity Analysis by Region

**Figure A1:**
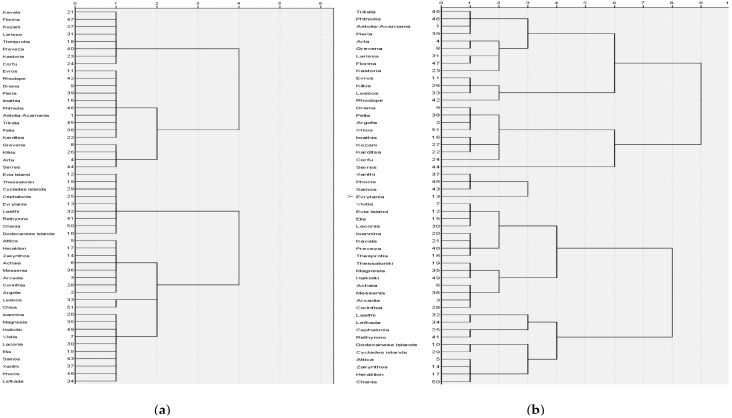
Cluster analysis. Dendrograms of regions by (**a**) quantities and (**b**) values.

Figure A1 at Appendix A presents the dendrograms of the cluster analysis. According to each cluster’s results and agglomeration schedule, we defined seven clusters for quantity (CCC = 75.6%) and nine for value (CCC = 83.1%). Table 6 presents the classification of the regions.

Looking at the results for the average amounts per inhabitant, gender, and drug category, some geographical similarities emerged in the clusters. Cluster 2 consists of areas of the Peloponnese and Southern Greece. There are three central prefectures (Achaia, Attica, and Heraklion), and all the rest are neighboring regions. In Cluster 6, there are areas from northwestern Greece; in Cluster 5, islands and areas with difficulty in accessing medical services; and in Cluster 7, islands of the northern Aegean. The results, in terms of prescription volume, showed a similar behavior in regions where there is difficulty in accessing structures and doctors (islands and mountainous areas without a good road network). These regions were in the lowest 25% for consumption in terms of important ATC drug categories (A, C, L). One explanation may be moving to urban areas where they had easier access to medical services. Areas near Athens and other large urban centers of the country showed similar behavior.

The corresponding geographical adjacencies did not occur in the value analysis, as only the neighboring prefectures of central Greece are in Cluster 1. In Cluster 3, most areas belong to the Peloponnese and Halkidiki with Thessaloniki.

To examine the similarity of the two classifications, based on the classification of the areas by the quantity data, the authors checked the similarity of the classification of the areas based on the value analysis. According to the results, all the areas belonging to Cluster 1 in the quantity analysis joined clusters with common areas, and in the value analysis, the value cluster included 40% of the areas of quantity Cluster 1. In quantity Cluster 2, only 1 in 8 areas were classified into a value cluster that did not include other common areas. The highest common classification was 50% (4/8 in the same group). Quantity Cluster 3 had a corresponding percentage of 50% (2/4 areas). In quantity Cluster 4, 90% (9 out of 10 regions) went to value clusters, where there were common regions, with the 4 regions having the highest common classification. In quantity Cluster 5, the percentage was 67% (6 out of 9), with a maximum number of common areas of 3. Finally, in quantity Cluster 6, the percentage was at 100%, with all areas corresponding to the new classification into clusters of common areas. The maximum number was three common areas.

## 4. Discussion

Demographic and geographic characteristics play a significant role in shaping prescription patterns and drug expenditure. Factors such as age, gender, socioeconomic status, and regional healthcare practices influence the types of medications prescribed and their associated costs [3]. Socioeconomic factors, including income and education levels, also impact prescription behaviors, with deprived areas often experiencing higher rates of drug-related admissions and overdoses [37]. Geographic variations, such as urban versus rural settings, further contribute to disparities, with urban areas generally having better access to healthcare facilities and specialists, leading to different prescribing practices compared to rural regions [9]. Understanding these demographic and geographic influences is crucial for developing targeted strategies to ensure equitable access to medications and to optimize healthcare outcomes across diverse populations.

Although Greek healthcare policies have legislated many measures according to international practices, in the last 15 years after the signing of the MoU in 2010 and in light of fiscal consolidation, comprehensive healthcare reform has been implemented, aiming, among other things, to reduce waste, control expenditure, and increase the accountability and efficiency of the Greek pharmaceutical sector [38]. The Greek government primarily focused on applying cost-containment measures, such as flat decreases in pharmaceutical prices and the collection of rebates from pharmaceutical companies to achieve a fast reduction in pharmaceutical expenditure. The value of the medicines prescribed before the 2009 crisis was over EUR 6 billion, with over 80% covered by the EOPYY. In 2013, it was reduced to EUR 2.4 billion, a 53% decrease since 2009, and in 2022, it was estimated to be catching up to EUR 6 billion again. Almost half of this amount is covered by the Health Social Insurance Fund (EOPYY), and the other half is covered by out-of-pocket payments (OOP) and pharmaceutical companies. OOP are about EUR 1 billion, and the pharmaceutical companies cover EUR 2 billion, through clawbacks and rebate mechanisms.

European states with similar compensation systems to Greece during the same period had small annual increases. France had an increase of 0.5% for the period 2010–2016 with an increase to 2% for the period 2017–21; Germany had an increase of 3.9% for the period 2010–2021; and Spain, a country that also signed an MoU, had a decrease in the period 2010–14, then a significant increase until 2016, and then, an increase of about 2.5% [39]. Italy, the UK, and the USA, which have different health systems from Greece, but similar OOP payment rates, had bigger increases, which exceeded those of Greece [6,39,40].

To the best of our knowledge, this is the first local study that showed the need for healthcare reforms to promote generic drugs. From surveys that studied the opinions of patients [41,42], doctors, and pharmacists [43], distrust from both patients and doctors was observed toward the effectiveness of generic drugs and the appropriateness of the regulatory authorities’ quality controls. Patient trust in doctors and pharmacists can dispel doubts about generic drugs. We believe that an information campaign on the efficacy and safety of generic drugs is necessary, as well as the establishment of incentives for doctors (general or internal medicine and cardiologists, who prescribe the majority of drugs) and pharmacists to promote them, alongside financial incentives given to patients [33]. At a later stage, following the practices of EU states [44] and other countries [45], it would be useful to design policies at the regional level.

Much of the expenditure is due to new high-value drugs, mainly in group L. It would be useful to consider other reimbursement models, such as risk-sharing contracts.

Moreover, our analysis showed that the 60+ age group had the highest percentage in consumption and expenditure on medicines, with the percentage remaining constant without changing from year to year of analysis. However, the younger age groups presented a significant annual increase in consumption and expenditure on medicines. This may be a consequence of the economic crisis and the pandemic and requires the state to take disease prevention measures. These findings may highlight the need for a comprehensive campaign focusing on preventive screening and lifestyle modifications. This initiative aims to enhance overall wellbeing by encouraging the early detection of health issues and fostering healthier habits.

An important finding in the research was the geographical separation of regions according to prescription and demographic data. Areas with more difficult access to health services showed different behaviour from the rest of the country, as did the residents of large urban centres. It would be useful to design policies specific to each region.

The time of the study also includes the 2020–21 period characterized by the COVID-19 pandemic. The consequences of the pandemic on prescriptions worldwide were an increase in expenditure [7,46], in particular, for specific categories of drugs such as anti-infectives for systemic use (the ATC J category) [16,47], in the period before the first vaccines were launched. In Greece, the increase in spending in 2020 was 7.7%, and in 2021, it was 5.3%. The use of anti-infectives for systemic use increased—in terms of quantity and value—significantly in 2020 but decreased in 2021. There was also a significant increase in preparations for the respiratory system (the ATC R category).

In addition, the results of the present study are the beginning of an investigation into variations in prescription characteristics. Since it is an important tool for the EOPYY (the Greek National Organization for the Provision of Health Services), policymakers and researchers must start a discussion about a real clinical and financial audit, combined with protocols for physicians and volume agreements with pharmaceutical companies that favour the healthcare system and, specifically, the patients.

Several potential limitations to this study must be considered. Firstly, we only included in our analysis drugs that are completely reimbursed by the EOPPY (another billion EUR was consumed by NHS hospitals: half of this was covered by the state and the other half by claw backs). Secondly, with the information about medications that is available in the administrative database, we were not able to identify any possible loss to follow-ups, making it impossible to assess the patients’ adherence to therapy.

It would be useful for subsequent research to address these limitations. It would also be useful for a study to examine the effect of new pharmaceuticals on costs.

## 5. Conclusions

The study highlights significant growth in prescription drug consumption and expenditure, with notable increases driven by demographic factors, regional disparities, and the introduction of high-cost drugs. Despite the establishment of an electronic system and the rise in generic drugs, particularly in cardiovascular treatments, overall spending continues to climb, suggesting a need for targeted cost-control measures. The study findings underscore the importance of addressing regional and demographic disparities in pharmaceutical use, thereby contributing to more equitable and cost-effective healthcare strategies. Moreover, the current study can provide a strategic framework for policymakers and healthcare managers to guide future studies and inform decision-making processes. It should be repeated every year going forward in order to succeed the above and expand scientific findings.

## Figures and Tables

**Table 1:** Quantity (volume) and expenditure by year, 2015–2021.

Measure	Year							Average
	2015	2016	2017	2018	2019	2020	2021	
Volume								
Total consumption (×1,000,000)	202.6	207.22	213.9	218.97	224.49	235.58	244.01	
Annual rate		2.6%	3.2%	2.4%	2.5%	4.9%	3.6%	3.1%
Consumption per capita	18.61	19.22	19.86	20.39	20.93	21.98	22.85	
Change rate		3.3%	3.4%	2.6%	2.7%	5.00%	4.00%	3.4%
Expenditure								
Total expenditure (×1,000,000 EUR)	3526.39	3449.34	3589.63	3830.06	4105.73	4421.99	4654.50	
Annual rate		−2.2%	4.1%	6.7%	7.2%	7.7%	5.3%	5.1%
Expenditure per capita (EUR)	324.77	319.86	333.36	356.58	382.83	412.55	435.87	
Change rate		−1.5%	4.2%	7.00%	7.4%	7.8%	5.7%	4.9%

**Table 2:** Trend analysis for volume and expenditure.

Variable	Coef.	Std. Err.	t	*p* > |t|	95% CI	
Volume						
Intercept	18.48	0.14	136.81	<0.001	18.13	18.83
Year	0.69	0.04	18.40	<0.001	0.60	0.79
Expenditure						
Intercept	305.67	8.53	35.82	<0.001	283.73	327.61
Year	20.29	2.37	8.57	<0.001	14.21	26.38

**Table 3:** Percentage of expenditure for generic drugs by category ATC-1 and year, 2015–21.

ATC-1 Group ^1^	2015	2016	2017	2018	2019	2020	2021	2015–2021
A	15.8%	16.7%	15.7%	17.1%	17.4%	17.5%	19.5%	17.3%
B	15.3%	15.9%	13.1%	11.7%	11.1%	10.6%	10.6%	12.2%
C	24.1%	24.8%	23.4%	24.5%	28.0%	31.6%	33.4%	27.0%
D	32.0%	38.3%	37.3%	35.6%	35.3%	35.4%	38.7%	36.2%
G	19.7%	20.2%	18.1%	17.6%	17.7%	18.1%	20.1%	18.8%
H	11.6%	14.2%	15.8%	17.1%	19.0%	20.3%	27.2%	18.5%
J	11.9%	9.6%	8.5%	9.1%	9.8%	8.4%	10.2%	9.5%
L	4.8%	5.4%	5.0%	4.6%	4.3%	3.9%	4.2%	4.5%
M	24.0%	22.5%	20.1%	18.7%	17.9%	18.0%	19.1%	20.0%
N	21.6%	23.5%	24.0%	25.6%	28.0%	28.9%	30.6%	26.1%
P	0.0%	0.0%	0.0%	2.4%	11.9%	9.7%	8.4%	5.7%
R	23.1%	21.7%	21.6%	20.5%	19.9%	18.6%	15.4%	19.9%
S	5.9%	8.9%	9.8%	11.7%	12.6%	13.8%	14.1%	10.8%
V	1.8%	4.1%	5.8%	6.6%	7.6%	9.8%	11.3%	7.0%
Total	16.1%	16.9%	15.4%	15.2%	15.7%	15.9%	16.8%	16.0%

^1^ A: alimentary tract and metabolism; B: blood and blood-forming organs; C: cardiovascular system; D: dermatology; G: genito-urinary system and sexual hormones; H: systemic hormonal preparations; J: anti-infectives for systemic use; L: antineoplastic and immunomodulating; M: musculo-skeletal system; N: nervous system; P: antiparasitic products; R: respiratory system; S: sensory organs; V: various.

**Table 4:** (**a**) Consumption distribution of the generic drugs prescription by category ATC-1 and age group, 2015–21. (**b**) Expenditure distribution of the generic drugs prescription by category ATC-1 and age group, 2015–21.

**(a)**					
**ATC-1**	**Consumption**				
**Group ^1^**	**0–14**	**15–29**	**30–44**	**45–59**	**60+**
A	1.70%	10.40%	15.30%	16.60%	17.10%
B	2.40%	12.40%	20.90%	8.80%	9.70%
C	0.30%	1.40%	5.50%	25.30%	38.40%
D	2.00%	8.90%	2.50%	1.30%	0.50%
G	0.00%	2.50%	6.10%	2.00%	2.40%
H	3.00%	3.70%	2.80%	2.80%	2.10%
J	62.30%	27.10%	12.60%	7.60%	3.90%
L	0.70%	1.90%	2.30%	2.50%	1.10%
M	2.60%	3.00%	3.40%	4.20%	3.60%
N	2.20%	13.60%	19.40%	21.30%	14.70%
P	0.10%	0.50%	0.60%	0.50%	0.20%
R	21.60%	12.50%	6.10%	5.10%	4.10%
S	0.80%	0.80%	0.50%	0.70%	1.80%
V	0.20%	1.30%	1,.80%	1.30%	0.50%
% to age	13.50%	29.10%	28.70%	28.20%	27.60%
% to pharma type	0.90%	2.20%	5.70%	13.10%	78.10%
**(b)**					
**ATC-1**	**Expenditure**				
**Group ^1^**	**0–14**	**15–29**	**30–44**	**45–59**	**60+**
A	1.40%	7.50%	8.80%	12.50%	16.00%
B	1.40%	12.00%	13.00%	6.90%	12.60%
C	0.60%	1.00%	3.40%	12.30%	22.00%
D	0.60%	3.70%	1.10%	0.60%	0.30%
G	0.00%	0.80%	3.80%	1.30%	1.90%
H	3.60%	7.40%	1.90%	1.80%	1.70%
J	75.50%	23.60%	10.80%	7.40%	4.40%
L	4.90%	20.00%	31.50%	33.20%	19.50%
M	0.70%	1.10%	1.20%	2.00%	3.00%
N	1.20%	9.30%	13.80%	12.70%	10.20%
P	0.00%	0.10%	0.10%	0.10%	0.10%
R	9.30%	10.50%	6.20%	5.50%	5.60%
S	0.20%	0.20%	0.20%	0.40%	1.40%
V	0.50%	2.70%	4.20%	3.20%	1.20%
% to age	4.70%	11.40%	13.10%	14.10%	17.40%
% to pharma type	0.70%	2.30%	6.30%	14.80%	75.90%

^1^ A: alimentary tract and metabolism; B: blood and blood-forming organs; C: cardiovascular system; D: dermatology; G: genito-urinary system and sexual hormones; H: systemic hormonal preparations; J: anti-infectives for systemic use; L: antineoplastic and immunomodulating; M: musculo-skeletal system; N: nervous system; P: antiparasitic products; R: respiratory system; S: sensory organs; V: various.

**Table 5:** (**a**) Consumption per capita by region and ATC-1, 2015–21. (**b**) Expenditure per capita by region and ATC-1, 2015–21.

**(a)**															
**Consumption**	**ATC ^1^**														
	**A**	**B**	**C**	**D**	**G**	**H**	**J**	**L**	**M**	**N**	**P**	**R**	**S**	**V**	**Total**
Eastern Macedonia and Thrace	3.2	1.9	7.0	0.2	0.5	0.4	1.2	0.2	0.8	3.1	0.0	0.9	0.2	0.1	19.7
Attica	3.0	1.8	5.5	0.2	0.4	0.4	1.2	0.3	0.6	2.9	0.0	0.9	0.3	0.1	17.5
North Aegean	3.8	2.0	6.7	0.2	0.5	0.6	1.4	0.3	0.9	3.0	0.1	1.0	0.4	0.1	21.0
Western Greece	3.0	1.8	6.1	0.2	0.4	0.4	1.3	0.2	0.7	3.0	0.1	1.0	0.4	0.1	18.7
Western Macedonia	3.4	2.1	7.6	0.2	0.6	0.5	1.3	0.3	0.8	3.1	0.1	0.9	0.2	0.1	21.1
Epirus	4.7	3.0	10.1	0.3	0.7	0.6	1.9	0.3	1.1	4.2	0.1	1.4	0.4	0.2	29.0
Thessaly	2.9	1.9	6.5	0.2	0.5	0.4	1.2	0.2	0.8	2.8	0.0	0.9	0.3	0.1	18.7
Ionian Islands	3.6	2.0	7.0	0.2	0.5	0.5	1.5	0.3	0.8	3.2	0.1	1.0	0.4	0.1	21.1
Central Macedonia	2.7	1.7	5.8	0.2	0.4	0.4	1.1	0.2	0.7	2.6	0.0	0.7	0.2	0.1	16.7
Crete	3.0	1.8	5.4	0.2	0.5	0.4	1.3	0.3	0.6	2.6	0.1	1.0	0.3	0.1	17.6
South Aegean	2.0	1.2	3.9	0.1	0.3	0.3	0.8	0.2	0.4	1.5	0.0	0.6	0.2	0.1	11.4
Peloponnese	3.2	1.8	5.9	0.2	0.5	0.5	1.3	0.2	0.7	2.9	0.0	1.1	0.3	0.1	18.6
Central Greece	2.8	1.8	5.9	0.2	0.4	0.4	1.1	0.2	0.7	2.7	0.0	0.9	0.3	0.1	17.5
Total	3.0	1.8	6.0	0.2	0.4	0.4	1.2	0.2	0.7	2.8	0.0	0.9	0.3	0.1	18.0
**(b)**															
**Expenditure (EUR)**	**ATC**														
	**A**	**B**	**C**	**D**	**G**	**H**	**J**	**L**	**M**	**N**	**P**	**R**	**S**	**V**	**Total**
Eastern Macedonia and Thrace	51.6	39.6	63.1	1.9	6.9	4.9	23.1	57.6	8.2	31.9	0.2	17.9	2.9	4.8	314.6
Attica	46.1	35.2	53.3	1.9	5.4	6.9	29.7	85.0	8.4	36.2	0.2	19.4	3.5	6.5	337.8
North Aegean	53.2	35.8	57.1	1.6	5.7	7.0	23.1	59.2	9.4	30.1	0.2	18.0	4.1	4.8	309.5
Western Greece	48.7	37.8	64.0	2.2	5.9	7.4	27.6	68.3	10.5	38.1	0.3	23.1	4.4	6.7	344.9
Western Macedonia	47.9	40.3	66.6	1.9	6.9	6.6	24.4	71.1	8.8	32.6	0.2	17.7	3.1	4.9	332.8
Epirus	45.7	37.7	70.1	1.9	5.6	5.5	24.0	62.8	9.7	36.7	0.2	21.5	3.5	4.2	329.2
Thessaly	45.1	40.7	64.7	1.9	6.6	6.6	26.0	67.6	9.2	34.8	0.2	21.4	3.9	6.6	335.2
Ionian Islands	49.1	35.2	66.4	1.7	6.0	6.4	26.8	75.5	9.2	37.2	0.2	21.1	4.3	5.5	344.5
Central Macedonia	45.8	40.9	58.3	2.1	6.4	6.6	26.7	70.8	8.3	32.7	0.2	15.8	2.8	6.0	323.5
Crete	44.6	32.1	50.4	1.8	6.4	8.1	28.2	72.5	8.1	36.8	0.5	23.6	3.9	5.0	322.2
South Aegean	44.3	27.1	47.9	1.6	4.5	5.7	25.4	69.0	6.5	23.6	0.1	14.7	3.1	4.5	278.2
Peloponnese	48.2	36.2	54.8	1.7	5.7	6.7	24.2	67.6	8.9	34.6	0.2	21.0	3.8	5.1	318.9
Central Greece	44.6	33.1	57.9	1.6	4.8	5.6	22.2	59.9	7.9	33.2	0.2	17.7	3.2	5.2	297.3
Total	46.6	36.7	57.3	1.9	5.9	6.6	27.0	73.6	8.6	34.6	0.2	19.2	3.5	5.9	327.5

^1^ A: alimentary tract and metabolism; B: blood and blood-forming organs; C: cardiovascular system; D: dermatology; G: genito-urinary system and sexual hormones; H: systemic hormonal preparations; J: anti-infectives for systemic use; L: antineoplastic and immunomodulating; M: musculo-skeletal system; N: nervous system; P: antiparasitic products; R: respiratory system; S: sensory organs; V: various.

**Table 6:** Cluster analysis: groups of regions.

Region	Cluster Number		Region	Cluster Number	
	Quantity	Expenditures		Quantity	Expenditures
Aetolia-Acarnania	1	1	Lefkada	4	8
Drama	1	2	Magnesia	4	3
Evros	1	6	Fokitha	4	7
Imathia	1	2	Samos	4	7
Karditsa	1	2	Viotia	4	5
Pella	1	2	Xanthi	4	7
Fhiotis	1	1	Kefalonia	5	8
Pieria	1	1	Chania	5	4
Rhodope	1	6	Cyclades islands	5	4
Trikala	1	1	Dodecanese Islands	5	4
Achaia	2	3	Evia Island	5	5
Arcadia	2	3	Evrytania	5	7
Argolis	2	2	Lasithi	5	8
Attica	2	4	Rethymno	5	8
Corinthia	2	3	Thessaloniki	5	3
Heraklion	2	4	Corfu	6	2
Messenia	2	3	Florina	6	1
Zakynthos	2	4	Kastoria	6	1
Arta	3	1	Kavala	6	5
Grevena	3	1	Kozani	6	2
Kilkis	3	6	Larissa	6	1
Serres	3	9	Preveza	6	5
Elis	4	5	Thesprotia	6	5
Halkidiki	4	3	Chios	7	2
Ioannina	4	5	Lesbos	7	6
Laconia	4	5			

## Data Availability

The data were provided by administrative sources only for the preparation of this research, without the right to grant them.
